# Efficient Degradation of Zearalenone by Dye-Decolorizing Peroxidase from *Streptomyces thermocarboxydus* Combining Catalytic Properties of Manganese Peroxidase and Laccase

**DOI:** 10.3390/toxins13090602

**Published:** 2021-08-28

**Authors:** Xing Qin, Yanzhe Xin, Xiaoyun Su, Xiaolu Wang, Yaru Wang, Jie Zhang, Tao Tu, Bin Yao, Huiying Luo, Huoqing Huang

**Affiliations:** State Key Laboratory of Animal Nutrition, Institute of Animal Sciences, Chinese Academy of Agricultural Sciences, Beijing 100193, China; qinxing@caas.cn (X.Q.); 18846920135@163.com (Y.X.); suxiaoyun@caas.cn (X.S.); wangxiaolu@caas.cn (X.W.); wangyaru@caas.cn (Y.W.); zhangjie09@caas.cn (J.Z.); tutao@caas.cn (T.T.); yaobin@caas.cn (B.Y.)

**Keywords:** dye-decolorizing peroxidase, mycotoxin, zearalenone, degradation, mediator

## Abstract

Ligninolytic enzymes, including laccase, manganese peroxidase, and dye-decolorizing peroxidase (DyP), have attracted much attention in the degradation of mycotoxins. Among these enzymes, the possible degradation pathway of mycotoxins catalyzed by DyP is not yet clear. Herein, a DyP-encoding gene, *St*DyP, from *Streptomyces thermocarboxydus* 41291 was identified, cloned, and expressed in *Escherichia coli* BL21/pG-Tf2. The recombinant *St*DyP was capable of catalyzing the oxidation of the peroxidase substrate 2,2′-azino-bis(3-ethylbenzothiazoline-6-sulfonic acid), phenolic lignin compounds 2,6-dimethylphenol, and guaiacol, non-phenolic lignin compound veratryl alcohol, Mn^2+^, as well as anthraquinone dye reactive blue 19. Moreover, *St*DyP was able to slightly degrade zearalenone (ZEN). Most importantly, we found that *St*DyP combined the catalytic properties of manganese peroxidase and laccase, and could significantly accelerate the enzymatic degradation of ZEN in the presence of their corresponding substrates Mn^2+^ and 1-hydroxybenzotriazole. Furthermore, the biological toxicities of the main degradation products 15-OH-ZEN and 13-OH-ZEN-quinone might be remarkably removed. These findings suggested that DyP might be a promising candidate for the efficient degradation of mycotoxins in food and feed.

## 1. Introduction

Zearalenone (ZEN) is a macrocyclic β-resorcylic acid lactone produced by *Fusarium* species, including *Fusarium graminearum*, *Fusarium culmorum*, *Fusarium equiseti*, and *Fusarium verticillioides* [1]. It is one of the most common mycotoxins, widely distributed in contaminated agricultural products and food stuffs such as maize, wheat, barley, and oats [2,3]. Due to its high affinity binding of the estrogenic receptors, ZEN and its metabolites exhibit potent reproductive toxicity, hepatotoxicity, hematotoxicity, immunotoxicity, and genotoxicity in humans and animals [4]. Generally speaking, large-scale food and feed contamination with ZEN could not only result in great economic loss, but also bring about a significant health threat to humans and animals. Moreover, ZEN is extremely heat-stable and resistant to conventional degradation methods such as physical and chemical approaches [5]. Therefore, there is an urgent need to establish efficient mycotoxin degradation strategies.

Recently, the biological degradation of different types of mycotoxins by ligninolytic enzymes, including laccase, manganese peroxidase, and dye-decolorizing peroxidase (DyP), has attracted more and more research attention because of its eco-sustainability and high efficiency [6,7,8,9,10]. It is worth mentioning that the catalytic mechanisms of various ligninolytic enzymes involved in mycotoxins degradation are quite different. For example, laccase can not only directly degrade aflatoxin B_1_, but also enhance the degradation of aflatoxin B_1_ in the presence of mediator syringaldehyde [6,8]. Manganese peroxidase is able to efficiently degrade multiple major mycotoxins, including aflatoxin B_1_, ZEN, deoxynivalenol, and fumonisin B1, through the action of Mn^3+^—derived radicals [9]. In contrast, the possible degradation mechanism and pathway of mycotoxins catalyzed by DyP are not yet clear, though DyP has been reported to degrade multiple major mycotoxins [10].

*Streptomyces* species are well-known for their efficient lignin degradation ability among lignin degrading bacteria [11,12]. Genome-wide analysis reveals that there are abundant lignin degrading related enzymes responsible for lignin depolymerization and utilization [13]. Laccase, multicopper oxidase, lignin peroxidase, and DyP consist of the ligninolytic enzyme system in *Streptomyces* species [14,15,16]. Herein, a novel dye-decolorizing peroxidase, *St*DyP, from *S. thermocarboxydus* 41291 was identified, cloned, expressed, and characterized. Moreover, the ZEN degrading ability of recombinant *St*DyP was evaluated. Furthermore, the possible degradation mechanism on the efficient degradation of ZEN by *St*DyP and the biological toxicities of the main degradation products were elucidated.

## 2. Results and Discussion

### 2.1. Cloning and Sequence Analysis of StDyP

Genome-wide annotation analysis indicated that the ligninolytic enzyme system of *S. thermocarboxydus* 41291 was comprised of two DyPs and two multicopper oxidases. One novel dye-decolorizing peroxidase-encoding gene, *St*DyP, was cloned from the genome of *S. thermocarboxydus* 41291. It consisted of an open reading frame of 1278 bp encoding 425 amino acid residues (Figure S1). The deduced *St*DyP harbored a twin-arginine translocase (TAT) signal peptide of 53 amino acid residues, which was commonly discovered in DyPs [17]. According to BLAST search result, the amino acid sequence of *St*DyP displayed the highest amino acid identity (43%) with previously identified DyP from *Thermobifida fusca* YX (Figure S2) [18]. Meanwhile, multiple sequence alignment revealed that *St*DyP contained the typical GXXDG motif (GQVDG) in the primary sequence, confirming that *St*DyP was a member of the DyP-type peroxidase family [19]. Based on the primary structural homology, the DyP-type peroxidase family was further categorized into four distinct classes: A, B, C, and D [20]. Class A DyP-type peroxidases contained a typical TAT signal peptide, exporting them into periplasmic space or extracellular space. In contrast, DyP-type peroxidases belong to class B and C were putative cytoplasmic enzymes. Class D DyP-type peroxidases were exclusively originated from fungi. The phylogenetic analysis exhibited that *St*DyP was closer to *Bp*hyDyPrx01, belonging to class A DyP-type peroxidases.

### 2.2. Expression and Purification of Recombinant StDyP

**Figure 1:**
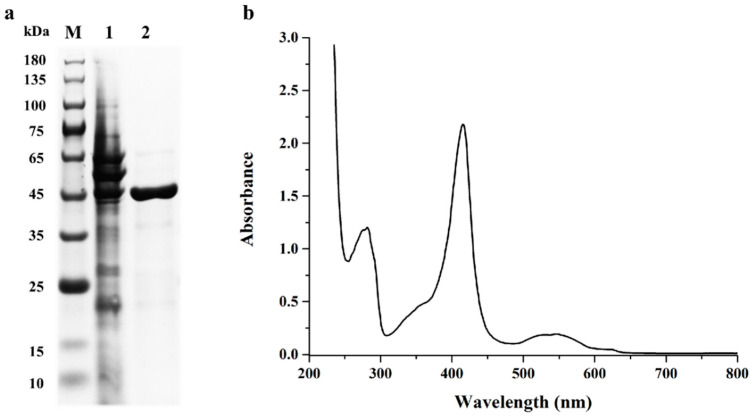
The analysis of purified recombinant *St*DyP by SDS-PAGE (**a**) and UV-visible spectroscopy (**b**). Lanes: M, the protein molecular mass marker; 1, the whole-cell lysis protein; 2, the purified recombinant *St*DyP containing a His-tag.

It was reported that using the cold shock-inducible expression system, coupled with co-expression of chaperones, was an efficient strategy for soluble expression of peroxidase in *E. coli* [10,21,22]. Therefore, the *St*DyP was cloned into the cold shock vector pCold I, resulting in the recombinant expression plasmid pCold I-*St*DyP (Figure S3), and the plasmid was subsequently transformed into the chaperone competent cell pG-Tf2/BL21. After the cells were harvested and disrupted by sonication, there was a remarkable peroxidase activity in the cell lysate supernatant, indicating that *St*DyP was expressed as a soluble form. The cell lysate supernatant was then applied onto an immobilized affinity chromatography containing nickel column for purification. SDS-PAGE analysis of the purified recombinant *St*DyP is shown in Figure 1a. The molecular weight of recombinant *St*DyP was estimated to be 43 kDa, which was similar to its calculated molecular mass.

### 2.3. Biochemical Characterization of Purified Recombinant StDyP

As shown in the UV-visible absorption spectrum (Figure 1b), the purified recombinant *St*DyP contained a Soret band at 416 nm and two Q bands at 529 and 546 nm, which was in good agreement with features of compound II identified in DyPs [23]. The Reinheitszahl (Rz, A_416_/A_280_) value of the purified recombinant *St*DyP was 1.83, indicating that the purity of *St*DyP was homogenous as compared to Rz values from reported DyPs, which ranged from 1.5 to 2.5 [24,25,26].

**Figure 2:**
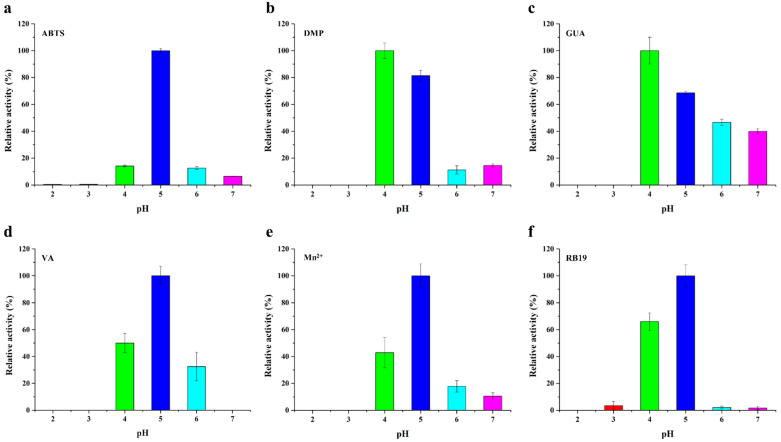
Optimum pH of the purified recombinant *St*DyP oxidizing different substrates: ABTS (**a**); DMP (**b**); GUA (**c**); VA (**d**); Mn^2+^ (**e**); and RB19 (**f**).

*St*DyP can catalyze the oxidation of various substrates, including the typical peroxidase substrate 2,2′-azino-bis (3-ethylbenzothiazoline-6-sulfonic acid) (ABTS), lignin model compounds 2,6-dimethylphenol (DMP), guaiacol (GUA) and veratryl alcohol (VA), manganese peroxidase substrate Mn^2+^, and anthraquinone dye reactive blue 19 (RB19) (Figure 2). Interestingly, *St*DyP exhibited Mn^2+^-oxidizing activity, which was thought to be the catalytic feature of manganese peroxidase and versatile peroxidase [27,28]. Moreover, *St*DyP was able to oxidize the non-phenolic substrate VA, which is the characteristic of high redox potential peroxidases such as lignin peroxidase and versatile peroxidase [29,30]. These results indicated that *St*DyP might have great potential for biodegradation due to its catalytic versatility.

The optimal pH for oxidation of various substrates by *St*DyP was different, exhibiting substrate-dependent optimum pH. The pH optimum for ABTS, VA, Mn^2+^ oxidation, and RB19 decolorization was 5, while optimal pH for DMP and GUA oxidation was found to be 4. In addition, the specific activity of *St*DyP for ABTS, DMP, GUA, VA, Mn^2+^, and RB19 at the corresponding optimal pH was 0.911 ± 0.016, 0.005 ± 0.000, 0.001 ± 0.000, 0.003 ± 0.000, 0.009 ± 0.001, and 0.127 ± 0.011 U/mg, respectively.

Like most DyP-type peroxidases belonging to class A, *St*DyP exhibits relatively low activity toward different types of substrates, including lignin model compounds [23]. Notably, *St*DyP can oxidize the non-phenolic substrate VA to veratraldehyde, while *Bs*DyP from *Bacillus subtilis* SCK6 and *Tc*DyP from *Thermomonospora curvata*, belonging to class A DyP-type peroxidases, cannot oxidize VA [10,23], which might be attributed to subtle differences in the position of the catalytic tryptophan residue. It was reported that the oxidizing capability of DyPs on the non-phenolic compound VA was affected by the side-chain orientation of a catalytic tryptophan [31]. Furthermore, compared with manganese peroxidases from white rot fungi [32,33], the specific activity of *St*DyP toward Mn^2+^ was more than three orders of magnitude lower due to the different binding modes of the divalent manganese ions between DyP and manganese peroxidase [34].

### 2.4. Enzymatic Degradation of ZEN by StDyP

**Figure 3:**
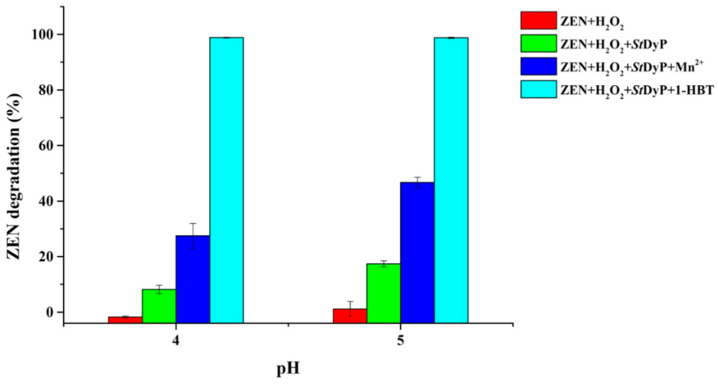
Degradation of ZEN by 1 U/mL *St*DyP in 50 mM malonate buffer (pH 4.0 and 5.0) containing 0.003 mM ZEN, 1 mM MnSO_4_ or 1-HBT, and 0.1 mM H_2_O_2_ at 30 °C for 48 h.

Ligninolytic enzymes, such as laccase, manganese peroxidase, and DyP, are known for their roles in the degradation of lignin and xenobiotic organic compounds [35]. Herein, the *St*DyP capability of degrading ZEN in the absence and presence of mediators was evaluated. As shown in Figure 3, direct degradation of ZEN by means of *St*DyP alone accounted for 8.17 ± 1.45% and 17.38 ± 1.09% degradation after 48 h reaction at pH 4 and 5, respectively. Moreover, it was noteworthy that the addition of the following mediators, Mn^2+^ or 1-HBT (1-hydroxybenzotriazole), could significantly enhance the degradation of ZEN. In the presence of Mn^2+^, the degradation percentage of ZEN by *St*DyP reached 27.49 ± 4.49% and 46.69 ± 1.85% at pH 4 and 5, respectively, which was consistent with characteristics of divalent manganese ions oxidation in *St*DyP. The improvement of ZEN degradation might be attributed to the functions of organic acid chelated Mn^3+^ and Mn^3+^-derived free radicals [9,10]. Moreover, our previous study demonstrated that Mn^3+^ and Mn^3+^-derived free radicals were involved in manganese peroxidase-catalyzed mycotoxin degradation [9]. In the case of 1-HBT, ZEN was almost completely degraded by *St*DyP at pH 4 and 5. This phenomenon was similar to the oxidation of the non-phenolic lignin model compound by the laccase/1-HBT system via generating a highly reactive nitroxyl radical [36]. However, the underlying mechanism of the DyP-catalyzed oxidation of 1-HBT for xenobiotic and lignin degradation has not yet been elucidated. To our knowledge, this was the first study concerning the efficient degradation of ZEN by DyP combining catalytic properties of manganese peroxidase and laccase, though the underlying mechanism of DyP-catalyzed ZEN degradation in the presence of 1-HBT remains to be revealed.

**Figure 4:**
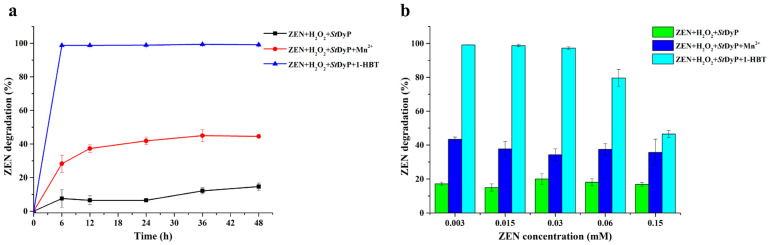
Time-course analysis of ZEN degradation by 1 U/mL *St*DyP in 50 mM malonate buffer (pH 5.0) containing 0.003 mM ZEN, 1 mM MnSO_4_ or 1-HBT, and 0.1 mM H_2_O_2_ at 30 °C (**a**). The effect of substrate concentration on the degradation of ZEN by 1 U/mL *St*DyP in 50 mM malonate buffer (pH 5.0) containing 0.003, 0.015, 0.03, 0.06, 0.15 mM ZEN, 1 mM MnSO_4_ or 1-HBT, and 0.1 mM H_2_O_2_ for 48 h at 30 °C (**b**).

Meanwhile, the time course of ZEN degradation by *St*DyP in the absence and presence of Mn^2+^ or 1-HBT was further assessed. In the presence of Mn^2+^, the degradation percentage of ZEN increased rapidly up to 37.28 ± 2.28% during the first 12 h, and then reached a plateau of approximately 45% after 36 h (Figure 4a). Significantly, the degradation of ZEN by *St*DyP in the presence of 1-HBT was a relatively rapid process, and 98.76 ± 0.20% of ZEN was removed after 6 h reaction. These differences could be ascribed to the oxidation capabilities of Mn^2+^ and 1-HBT by *St*DyP, particularly its low catalytic efficiency for Mn^2+^.

In addition, enzymatic degradation of ZEN at various initial concentration (0.003, 0.015, 0.03, 0.06, 0.15 mM ZEN) by *St*DyP was carried out at 30 °C for 48 h. There was no significant influence of initial concentration on the degradation percentage by *St*DyP in the absence and presence of Mn^2+^. However, in the presence of 1-HBT, *St*DyP showed decreased degradation percentage with increasing concentrations of ZEN. When the concentration of ZEN increased to 0.06 and 0.15 mM, the degradation percentage of ZEN decreased to 79.64 ± 5.01% and 46.50 ± 2.08%, respectively.

As for degradation of ZEN by enzymes, the lactonohydrolase ZHD101 from *Clonostachys rosea* was one of the most studied ZEN-degrading enzymes [37]. However, the maximal activity of ZHD101 was found at pH 10.5, which limited its applications in contaminated food and feed. Recently, our studies revealed that ligninolytic enzymes such as laccase, manganese peroxidase, and DyP were capable of degrading different types of mycotoxins in neutral and acidic conditions [8,9,10]. In comparison with previous studies, this work firstly demonstrated that DyP could significantly accelerate the enzymatic degradation of ZEN in the presence of Mn^2+^ and 1-HBT, providing another efficient approach for ZEN degradation by DyP.

### 2.5. Identification of ZEN Degradation Products

**Figure 5:**
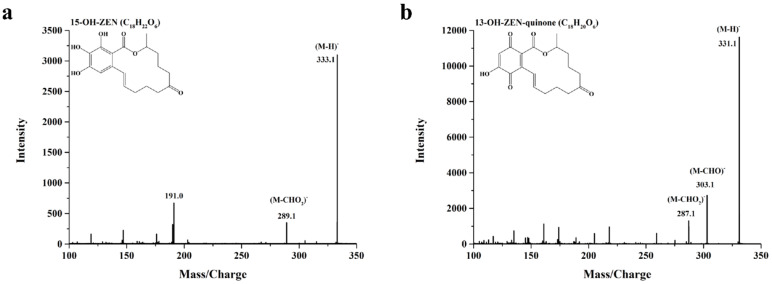
HPLC-MS/MS analysis of ZEN degradation products, including 15-OH-ZEN (**a**) and 13-OH-ZEN-quinone (**b**), by 1 U/mL *St*DyP in 50 mM malonate buffer (pH 5.0) supplemented with 1 mM MnSO_4_ or 1-HBT, and 0.1 mM H_2_O_2_ at 30 °C.

To further elucidate the degradation mechanism of ZEN by *St*DyP, the degradation products of ZEN by *St*DyP were characterized by HPLC-MS/MS. It was found that there was no difference in the types of the main degradation products among various ZEN-degradation systems, although there was a difference in quantity. As shown in Figure 5, two main degradation products were detected in the enzymatic reactions. One degradation product exhibited the parent ion at m/z 333.1 (M-H)^-^, producing daughter ions of 289.1 (M-44-H)^-^ and 191.0 (M-142-H)^-^ (Figure 5a). These daughter ions were in accordance with the MS/MS fragments of 15-OH-ZEN, corresponding to a formula of C_18_H_22_O_6_ [38]. The other degradation product showed the parent ion at m/z 331.1 (M-H)^-^, generating daughter ions at 303.1 (M-28-H)^-^ and 287.1 (M-44-H)^-^ (Figure 5b). These daughter ions were in good agreement with the MS/MS fragments of 13-OH-ZEN-quinone, corresponding to a formula of C_18_H_20_O_6_ [38,39]. In comparison with the amount of the degradation product 15-OH-ZEN, 13-OH-ZEN-quinone in various ZEN-degradation systems accumulated at a relatively high amount (Figure S4).

Based on the structures of the main degradation products, the putative degradation mechanism of ZEN by *St*DyP was proposed. The main degradation products, including 13-OH-ZEN and 15-OH-ZEN, were produced by hydroxylation of the aromatic moiety at position 13 and 15. However, 13-OH-ZEN was chemically unstable and prone to autoxidation to 13-OH-ZEN-quinone (Figure S5). Furthermore, the biological toxicities of the degradation products were analyzed based on the relationship between chemical structure and biological toxicity. Considering that the hydroxylation of the aromatic moiety in ZEN exhibited an obviously decreased estrogenicity [40], the biological toxicities of the main degradation products of ZEN by *St*DyP might be significantly removed. For example, the estrogenicity of 15-OH-ZEN had been reported to be decreased by 98% compared with ZEN [40].

## 3. Conclusions

In summary, a novel dye-decolorizing peroxidase-encoding gene, *St*DyP, from *S. thermocarboxydus* 41291 was identified, cloned, and expressed. *St*DyP could not only oxidize phenolic and non-phenolic lignin model compounds, but also decolorize the representative anthraquinone dye RB19. Moreover, *St*DyP could remarkably enhance the degradation of ZEN using the catalytic properties of manganese peroxidase and laccase. Furthermore, the biological toxicities of degradation products of ZEN by *St*DyP might be significantly removed. These findings suggested that using the catalytic versatility of DyP might be an efficient approach to degrade ZEN in contaminated food and feed.

## 4. Material and Methods

### 4.1. Strains and Substrates

*Streptomyces thermocarboxydus* 41291 was purchased from the Agriculture Culture Collection of China (Beijing, China) and was maintained at 4 °C on agar plate of modified Gause’s No. 1 medium (g/L): KNO_3_, 1.0; KH_2_PO4, 0.5; MgSO_4_, 0.5; FeSO_4_, 0.01; NaCl, 0.5; soluble starch, 20.0; agar 15.0; pH 7.2-7.4. *Escherichia coli* Trans1-T1 and pG-Tf2/BL21 were purchased from TransGen and Takara (Beijing, China), respectively. ABTS, DMP, GUA, VA, RB19, and ZEN were purchased from Sigma–Aldrich (St. Louis, MO, USA). All other chemicals were of analytical grade and commercially available.

### 4.2. Identification and Cloning of StDyP

The genomic DNA of *S. thermocarboxydus* 41291 was extracted using a TIANamp Bacteria DNA Kit according to the manufacturer’s instruction, and sequenced on an Illumina HiSeq platform using 150-bp paired-end sequencing to obtain a draft genome. All proteins encoded by the genome of *S. thermocarboxydus* 41291 were subjected to CAZymes annotation using the Hidden Markov Model from dbCAN. The enzymes in the CAZy family AA2 were further confirmed via a BLAST search against the RedoxiBase databases for DyPs annotation.

Based on the 5′- and 3′-end sequences of the *St*DyP structural gene, the *St*DyP gene devoid of the signal sequence was amplified with gene-specific primers from genomic DNA of *S. thermocarboxydus* 41291 (*St*DyP-*Nde*I-F: 5’ ATCATCATATCGAAGGTAGG*CATATG*CCCACCGGAGCCACTCCGCTCAC 3’; *St*DyP-*Xba*I-R: 5’ TTTTAAGCAGAGATTACCTA*TCTAGA*CCCCTCCAGCAGCGCCTGGCCC 3’). The PCR product was assembled into pCold I and transformed into the *E. coli* Trans1-T1 to construct a recombinant expression plasmid pCold I-*St*DyP. After the reading frame was confirmed as being correct by sequencing, the recombinant plasmid was transformed into the expression host *E. coli* pG-Tf2/BL21.

### 4.3. Expression and Purification of StDyP

The transformant harboring pCold I-*St*DyP was picked and cultured at 37 °C overnight in LB medium supplemented with 100 µg/mL ampicillin and 20 µg/mL chloramphenicol. The overnight culture was then inoculated into fresh LB medium containing 5 ng/mL tetracyclin, 100 μg/mL ampicillin, and 20 μg/mL chloramphenicol. When OD 600 of the culture reached 1.0, the culture was added to 0.5 mM isopropy-β-D-thiogalactoside (IPTG) and 20 μM hemin, followed by incubation for another 20 h at 16 °C.

The cells were harvested and resuspended in binding buffer (20 mM sodium phosphate, 500 mM NaCl, pH 7.4). After sonication on ice for 30 min, the resulting lysates were centrifugated to obtain crude protein mixtures. The recombinant *St*DyP was then purified from the mixtures by using immobilized affinity chromatography containing nickel column. The purity of recombinant *St*DyP was validated by SDS-PAGE using 10% polyacrylamide gel.

### 4.4. Biochemical Characterization of StDyP

The DyP activity was measured by monitoring the oxidation of ABTS at 420 nm in 50 mM malonate buffer (pH 5.0) containing 1 mM ABTS, 0.1 mM H_2_O_2_, and the appropriately diluted enzyme. One unit of enzyme activity was defined as the amount of enzyme that oxidized 1 μmol of ABTS per min at 25 °C.

The UV-visible spectroscopy analysis of purified recombinant DyP was measured in the range from 230 nm to 800 nm. The Rz value of *St*DyP was calculated as the ratio of absorbance due to hemin (A_416_, Soret region) to absorbance due to protein (A_280_).

The substrate specificity of *St*DyP was carried out for the oxidation of various substrates, including ABTS (ε_420_ = 36,000,000 mM^−1^·cm^−1^), DMP (ε_470_ = 12,100,000 mM^−1^·cm^−1^), GUA (ε_465_ = 49,600,000 mM^−1^·cm^−1^), VA (ε_310_ = 9,300,000 mM^−1^·cm^−1^), Mn^2+^ (ε_270_ = 11,590,000 mM^−1^·cm^−1^), and RB19 (ε_595_ = 10,000,000 mM^−1^·cm^−1^), in the pH range of 2.0 to 7.0 at 25 °C. The maximum activity of DyP was considered to be 100%.

### 4.5. Enzymatic Degradation of ZEN by StDyP

The ZEN degradation ability of *St*DyP was first evaluated in the presence of Mn^2+^ or 1-HBT. The degradation of ZEN was performed in 50 mM malonate buffer (pH 4.0 or 5.0) containing 0.003 mM ZEN, 1 mM MnSO_4_ or 1-HBT, 0.1 mM H_2_O_2_, and 1 U/mL *St*DyP for 48 h at 30 °C.

Secondly, the time course of ZEN degradation by *St*DyP was determined in 50 mM malonate buffer (pH 5.0) containing 0.003 mM ZEN, 1 mM MnSO_4_ or 1-HBT, 0.1 mM H_2_O_2_, and 1 U/mL *St*DyP for 6, 12, 24, 36, and 48 h at 30 °C. In addition, the effect of substrate concentration on the degradation of ZEN was assessed in 50 mM malonate buffer (pH 5.0) containing 0.003, 0.015, 0.03, 0.06 or 0.15 mM ZEN, 1 mM MnSO_4_ or 1-HBT, 0.1 mM H_2_O_2_, and 1 U/mL *St*DyP for 48 h at 30 °C.

HPLC analysis of ZEN degradation was performed by using a SHIMADZU 20A series instrument (Kyoto, Japan) equipped with a UV/Vis detector and RF-20A fluorescence detector with a Waters XBridge C18 column (5 µm, 4.6 mm × 150 mm). The elution condition for ZEN was set as follows: 45% acetonitrile (ACN) at a flow rate of 0.75 mL/min; ZEN was monitored at 274 nm excitation wavelength and 440 nm emission wavelength.

### 4.6. Identification of ZEN Degradation Products

ZEN degradation products were analyzed by using an AB-SCIEX 5600 Triple TOF Mass Spectrometer with Shimadzu Nexera Series Liquid Chromatograph employing a Waters XBridge BEH C18 column (2.5 µm, 2.1 × 150 mm) under negative ion and high-sensitivity mode. For LC analysis, the elution condition was 0–70% ACN, 7 min; 70–100% ACN, 5 min; and 100% ACN, 1 min at a flow rate of 0.3 mL/min. For MS analysis, the parameters were set as following: GS1, 55 psi; GS2, 55 psi; curtain gas, 25 psi; temperature, 500 °C; and ion spray voltage floating, 5500 V.

## Data Availability

The data presented in this study are available on request from the corresponding author.

## Supplementary Materials

The following are available online at https://www.mdpi.com/article/10.3390/toxins13090602/s1, Figure S1: The nucleotide and deduced amino acid sequence of *St*DyP from *Streptomyces thermocarboxydus*. Red box: the signal peptide; blue underline: the GXXDG motif. Figure S2: The amino acid sequence alignment of *St*DyP with the *Tf*DyP from *Thermobifida fusca* YX. Figure S3: The recombinant plasmid map of pColdI-*St*DyP. Figure S4: MS/MS spectra of ZEN degradation products by *St*DyP in the presence of Mn^2+^ or 1-HBT for 6 and 48 h. Figure S5: The possible ZEN degradation pathways by *St*DyP from *Streptomyces thermocarboxydus*.
