# Supplementary material for: Efficient Degradation of Zearalenone by Dye-Decolorizing Peroxidase from Streptomyces thermocarboxydus Combining Catalytic Properties of Manganese Peroxidase and Laccase
Source: Toxins (Basel). 2021 Aug 28;13(9):602. doi: 10.3390/toxins13090602 (PMC8473283; doi:10.3390/toxins13090602)
Supplement: Supplementary file 1 [file toxins-13-00602-s001.zip › toxins-1351255-supplementary.pdf]

Efficient Degradation of Zearalenone by Dye-Decolorizing Peroxidase from *Streptomyces Thermocarboxydus* Combining Catalytic Properties of Manganese Peroxidase and Laccase

Xing Qin, Yanzhe Xin, Xiaoyun Su, Xiaolu Wang, Yaru Wang, Jie Zhang, Tao Tu, Bin Yao, Huiying Luo and Huoqing Huang

```
1      ATGGCTGACCCTTCCCTGTCGCAGACCCGCACCCCCGAGAAGGAGCCCCAGGCGGAAGCC
1       M  A  D  P  S  L  S  Q  T  R  T  P  E  K  E  P  Q  A  E  A

61     GCCGCCTCCGGCATCTCGCGGCGGCGCCTGCTCGGCACGGCCGGCGCCACCGGGCTCGTG
21      A  A  S  G  I  S  R  R  R  L  L  G  T  A  G  A  T  G  L  V

121    CTCGGGGCGGCCGGCGGTGCCGTCGGGTACGCGTCGGCGCCCACCGGAGCCACTCCGCTC
41      L  G  A  A  G  G  A  V  G  Y  A  S  A  P  T  G  A  T  P  L

181    ACCTCGGTCGGCGCCACAAAAGTCCCGTTTCACGTGAAACATCAGCCGGGCATCACCGAC
61      T  S  V  G  A  T  K  V  P  F  H  V  K  H  Q  P  G  I  T  D

241    CCGCTCCAGTCGCGTGGCCATCTCCTCGCCTTCGACCTGAGGCCCGGCGCCGGACGCAAG
81      P  L  Q  S  R  G  H  L  L  A  F  D  L  R  P  G  A  G  R  K

301    GAGGCGGCTGCGCTGCTGCGCCGCTGGTCCGACACCGCCCGGCGGCTGATGGACGGGACG
101     E  A  A  A  L  L  R  R  W  S  D  T  A  R  R  L  M  D  G  T

361    TTCGACGCCGAGGGCGACAGTGACGTGGCCCGTGACGCGGGGCCCTCCTCGCTGACCCTG
121     F  D  A  E  G  D  S  D  V  A  R  D  A  G  P  S  S  L  T  L

421    ACCTTCGGTTTTGGGCACAGCTTCTTCGCGCGCACCGGGCTGGAGAGGCAGCGTCCGGCC
141     T  F  G  F  G  H  S  F  F  A  R  T  G  L  E  R  Q  R  P  A

481    GCCCTGGAGCCGCTGCCCGCCTTCTCCTCCGACCGCCTCGACCGGGCCCGCAGCGACGGG
161     A  L  E  P  L  P  A  F  S  S  D  R  L  D  R  A  R  S  D  G

541    GACCTGTGGGTGCAGATTGGCGCCGACGACGCCCTCGTCGCGTTCCATGCCCTGCGCGCG
181     D  L  W  V  Q  I  G  A  D  D  A  L  V  A  F  H  A  L  R  A

601    GTGCAGAAGGACGCGGGCGCGGCGGCCCGGGTGCGCTGGCAGATGAACGGCTTCAACCGG
201     V  Q  K  D  A  G  A  A  A  R  V  R  W  Q  M  N  G  F  N  R

661    TCGCCGGGCGCCACCGCCCGCCCGATGACCACCCGCAATCTGATGGGCCAGGTCGACGGC
```

```
221     S P G A T A R P M T T R N L M G Q V D G

721     ACCCGCAACCCGAAACCCGACGAGCCCGACTTCGACCAGCGGATCTTCGTGGCGGAGCAG
241     T R N P K P D E P D F D Q R I F V A E Q

781     GGCGAGCCCGCCTGGATGGCGAACGGCTCCTATGTGGTCGTCCGCCGGATCCGCATGCTG
261     G E P A W M A N G S Y V V V R R I R M L

841     CTGGACGACTGGGAGAAGCTGTCGCTCAGGGAGCAGGAGGGTGTCATCGGGCGGCGCAAG
281     L D D W E K L S L R E Q E G V I G R R K

901     GCGGACGGCGCCCCGCTCTCCGGGGGCGACGAGACGACCGAGATGGACCTGGAGAAGACC
301     A D G A P L S G G D E T T E M D L E K T

961     GACGCCCAGGGCAATCTGGTCGTCCCGTTCAACGCGCACGCACGCATCACCCGGCCCGAC
321     D A Q G N L V V P F N A H A R I T R P D

1021    CAGAACGGCGGGGCGGCGATGCTGCGCCGGCCGTTCTCGTACCACGACGGCATCGACGCG
341     Q N G G A A M L R R P F S Y H D G I D A

1081    GACGGGACGCCGGACGCGGGGCTGCTGTTCATCTGCTGGCAGGCCGATCCGCTGCGCGGC
361     D G T P D A G L L F I C W Q A D P L R G

1141    TTCGTGCCGGTGCAGCGCAAGCTCGACCGGGGCGACGCCCTGACGCCGTTCATCCGTCAC
381     F V P V Q R K L D R G D A L T P F I R H

1201    GAGGCGAGCGGGCTGTTCGCCGTCCCGGGCGGGGCGGCGGAGGGTGAGTATGTGGGCCAG
401     E A S G L F A V P G G A A E G E Y V G Q

1261    GCGCTGCTGGAGGGGTGA
421     A L L E G *
```

Figure S1. The nucleotide and deduced amino acid sequence of *St*DyP from *Streptomyces thermocarboxydus*. Red box: the signal peptide; blue underline: the GXXDG motif.

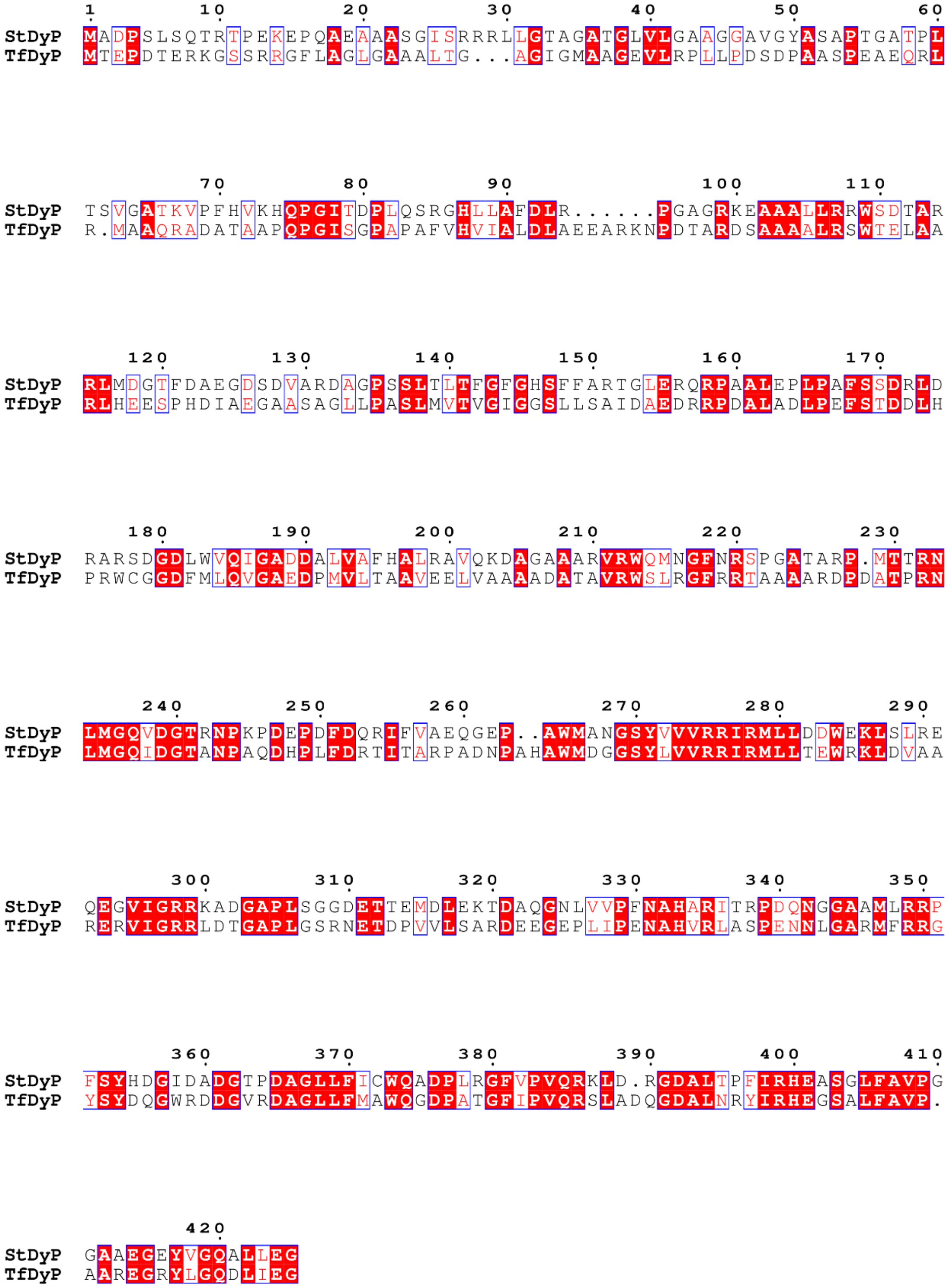

Figure S2. The amino acid sequence alignment of *St*DyP with the *Tf*DyP from *Thermobifida fusca*.

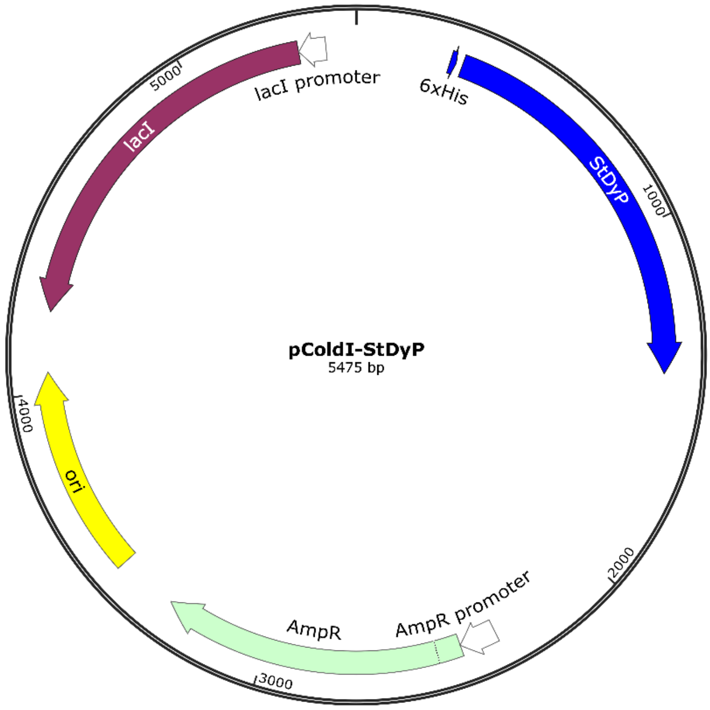

Figure S3. The recombinant plasmid map of pColdI-*St*DyP.

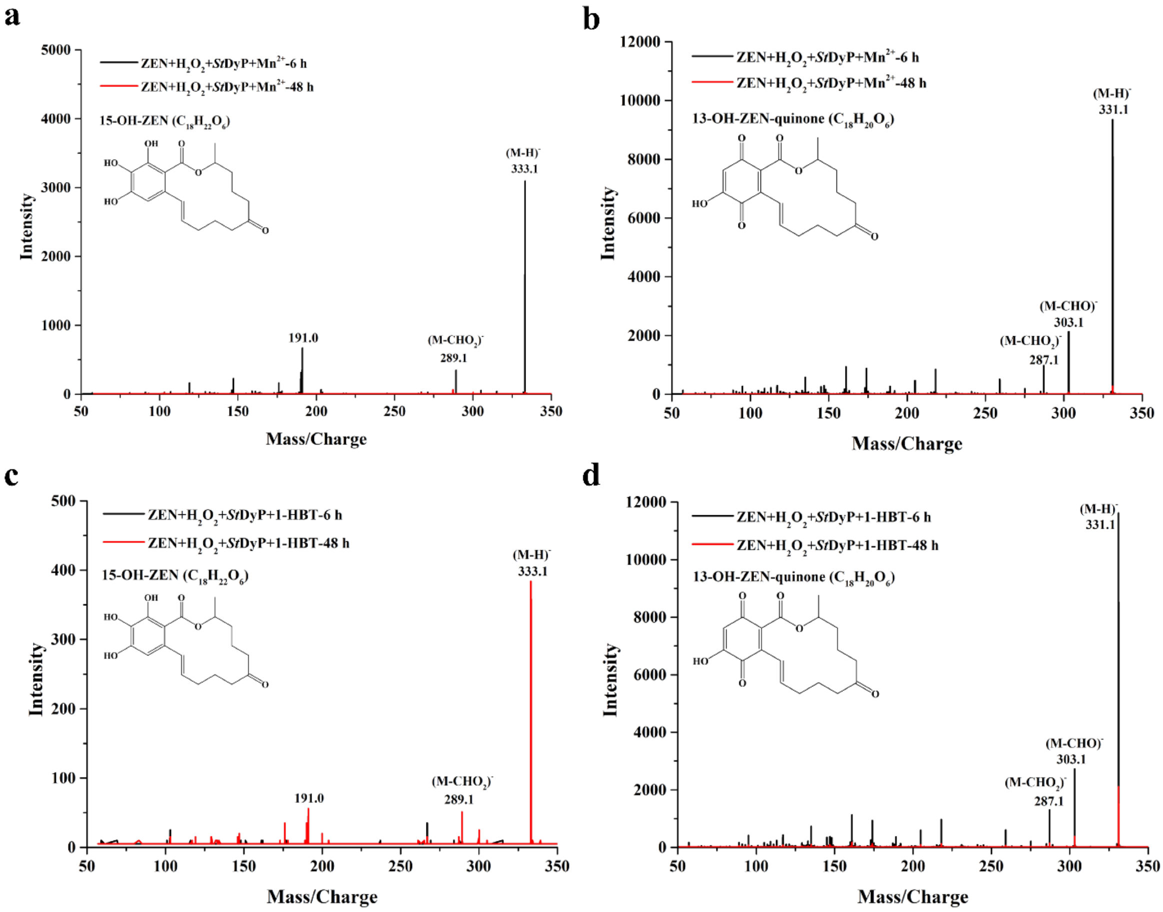

Figure S4. MS/MS spectra of ZEN degradation products, including 15-OH-ZEN and 13-OH-ZEN-quinone, by *St*DyP in the presence of Mn^{2+} (**a** and **b**) or 1-HBT (**c** and **d**) for 6 and 48 h.

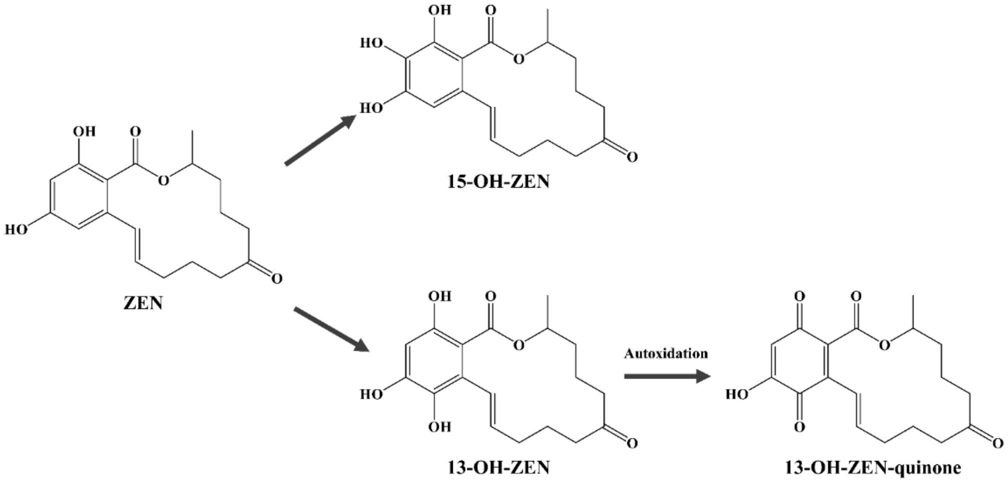

Figure S5. The possible ZEN degradation pathways by *St*DyP from *Streptomyces thermocarboxydus*.
